# Effects of Chinese Medicine on modulating interleukin-17-regulated macrophages in coronary heart disease

**DOI:** 10.3389/fphar.2025.1499786

**Published:** 2025-04-10

**Authors:** Qingqing Liu, Peizhong Liu, Chuangpeng Li, Zhen Zhao, Dawei Wang, Qing Liu, Huawei Yang

**Affiliations:** ^1^ Guangdong Provincial Hospital of Chinese Medicine‐Zhuhai Hospital, State Key Laboratory of Traditional Chinese Medicine Syndrome, The Second Affiliated Hospital of Guangzhou University of Chinese Medicine, Guangdong Provincial Hospital of Chinese Medicine, Guangzhou, Guangdong, China; ^2^ State Key Laboratory of Traditional Chinese Medicine Syndrome, The First Affiliated Hospital of Guangzhou University of Traditional Chinese Medicine, Guangzhou, Guangdong, China

**Keywords:** Chinese medicine (CM), interleukin-17 (IL-17), macrophages, coronary heart disease (CHD), Th17 cell

## Abstract

Coronary atherosclerotic heart disease (CHD) is one of the leading causes of death from cardiovascular disease worldwide and has significant inflammatory features. Macrophages play an important role in atherosclerotic plaque formation and inflammation. IL-17, as a pro-inflammatory cytokine, further exacerbates the development of CHD by interacting with macrophages. In recent years, there has been increasing evidence that traditional Chinese medicine (CM) has a wide range of applications in regulating the immune system and treating CHD. This article reviewed the role of CM in the regulation of IL-17-regulated macrophages, discussed the core components and targets of CM in the treatment of CHD, and laid a theoretical foundation for its clinical application. The results show that CM can effectively inhibit the formation of foam cells, stabilize vulnerable plaque and delay the progression of atherosclerosis by inhibiting inflammation, regulating the polarization of macrophages and promoting cholesterol outflow. In addition, CM can also regulate the expression and signaling pathway of IL-17, further inhibit inflammatory response and improve the symptoms of CHD, providing a new idea and method for the prevention and treatment of CHD.

## 1 Introduction

Coronary heart disease (CHD) is one of the most prevalent chronic cardiovascular diseases worldwide, posing a significant threat to human health due to its high incidence and mortality rates. The development of CHD is influenced by various factors, including genetics, lifestyle choices, and environmental conditions. In recent years, changes in lifestyle and an aging population have contributed to a rising trend in the incidence of CHD, making it a major challenge in the field of global public health. Abnormal lipid metabolism is the most important risk factor for atherosclerosis (AS), especially the increase in oxidized low-density lipoprotein (ox-LDL), leading to chemokines secretion and inflammation in endothelial and immune cells (Wu et al., 2017). Dendritic cells (DCs) and macrophages are important antigen-presenting cells (APCs) involved in antigen presentation and foam cells formation in the inflammatory process (Groh et al., 2018). T cells activated by APC secrete interleukin (IL)-17 and interferon-gamma (IFN-γ), resulting in AS plaques progression and platelet aggregation (Wu et al., 2017). During this process, lesion progression is mainly mediated by cells of the monocyte/macrophages spectrum. Furthermore, the presence of macrophages of different phenotypes (e.g., extreme phenotypes M1, M2) in plaques shows heterogeneity, including pro- and anti-inflammatory functions (Fernandez et al., 2019).

IL-17 is a potent pro-inflammatory cytokine that exacerbates the progression of CHD by interacting with macrophages. It promotes the migration of macrophages to the arterial wall, stimulates the release of inflammatory factors, and increases plaque susceptibility (Smith et al., 2010). These processes can ultimately lead to plaque rupture and cardiovascular events, such as heart attacks. Macrophages exist in various subpopulations with distinct functions, complicating the treatment of CHD. While pro-inflammatory M1 macrophages contribute to plaque instability, anti-inflammatory M2 macrophages may provide a protective effect. Understanding and regulating this balance is crucial for effective treatment. This article focuses on the role of IL-17-regulated macrophages in the pathophysiological mechanisms involved in CHD from both a clinical and experimental perspective. In addition, Chinese medicine (CM) has a wide range of applications in regulating the immune system. With the intensive research on CM pharmacology, more and more CM have been found to intervene and modulate the role of IL-17 and macrophages in CHD. The article will discuss how CM modulates the effect of IL-17-regulated macrophages on CHD. Additionally, Th17 cells and regulatory T cells (Tregs) are two key immune cell subpopulations that play an important role in immune balance. Th17 cells mainly secrete pro-inflammatory cytokines such as IL-17, while Treg cells secrete anti-inflammatory cytokines such as IL-10 and inhibit the activity of Th17 cells. Th17/Treg imbalance leads to an overactive inflammatory response, which promotes atherosclerosis and the development of CHD (Wang Q. et al., 2022).

## 2 CM modulates the pathogenic roles of macrophages in CHD development

### 2.1 Mechanism of macrophages in CHD development

The formation of AS is a complex multi-stage process in which macrophages play a crucial role. In the early stage, low-density lipoprotein (LDL) is oxidized in the subendothelial space to form oxidized low-density lipoprotein (ox-LDL). The presence of ox-LDL activates DCs and T cells, and promotes the differentiation of Th17 cells, resulting in the production of the pro-inflammatory cytokines IL-17 and IFN-γ (Wang Y. K. et al., 2022). Ox-LDL also triggers the expression of adhesion molecules, chemotactic cytokines, and pro-inflammatory factors in macrophages and vascular endothelial cells, leading to systemic and local immune responses. Macrophages, stimulated by macrophage colony-stimulating factor (GM-CSF) (Johnson and Newby, 2009), have a crucial role in AS lesions and switch between M1 and M2 phenotypes (Tacke and Zimmermann, 2014). To be specific, these cytokines activate macrophages, shifting them from the M0 to the pro-inflammatory M1 phenotype. M1 macrophages release pro-inflammatory factors like TNF-α, IL-6, and IL-1β, contributing to foam cell formation and accelerating atherosclerosis plaque development. As plaques progress, macrophage phenotype shifts, with M2 macrophages gradually increasing. M2 macrophages exhibit anti-inflammatory and tissue repair functions, secreting IL-10, TGF-β, and other anti-inflammatory factors, and participating in angiogenesis and matrix remodeling. They also contribute to reverse cholesterol transport, removing cholesterol from foam cells and reducing plaque burden. However, in the late stages of plaque development, M2 macrophages may secrete proteases such as MMPs, potentially leading to plaque instability and rupture.

Inflammatory mechanisms mediated by immune cells can directly impact plaque stability and trigger plaque rupture. Macrophages, located near lipid streaks or AS plaques, produce reactive oxygen species and proteases through scavenger receptors like type A scavenger receptor (SRA) and CD36. These receptors facilitate the phagocytosis of ox-LDL, leading to nonspecific immune responses and the formation of foam cells and lipid streaks (Kunjathoor et al., 2002). As lipids accumulate and vascular smooth muscle cells (VSMC) proliferate, AS plaques or intimal and intima-media hyperplasia form, narrowing the arteries and reducing blood flow to the heart. This leads to symptoms like chest pain, angina pectoris, and potentially myocardial infarction. Macrophages play a crucial role in every stage of this process, from endothelial dysfunction and lipid deposition to inflammation and plaque formation.

Pro-inflammatory macrophages count in perivascular adipose tissue (PvAT) inflammation (M1) and anti-inflammatory (M2) macrophages counts are closely associated with AS (Farias-Itao et al., 2022), and PvAT macrophages are associated with adjacent vessel stenosis (Verhagen et al., 2014). M2 macrophages are more abundant than M1 macrophages in PvAT. M1 macrophages are associated with coronary thrombosis in thrombotic plaques, while M2 macrophages are linked to a reduced number of epicardial vessels. Macrophages induce VSMC apoptosis through the FAO pathway and secretion of TNF-α and nitric oxide (NO). Furthermore, the Th17/Treg imbalance is also one of the crucial mechanisms in the development of CHD. The IL-17 secreted by Th17 cells further enhances the inflammatory response of macrophages, while Treg cells restrain the activity of Th17 cells, thereby suppressing the inflammatory response.

**FIGURE 1:**
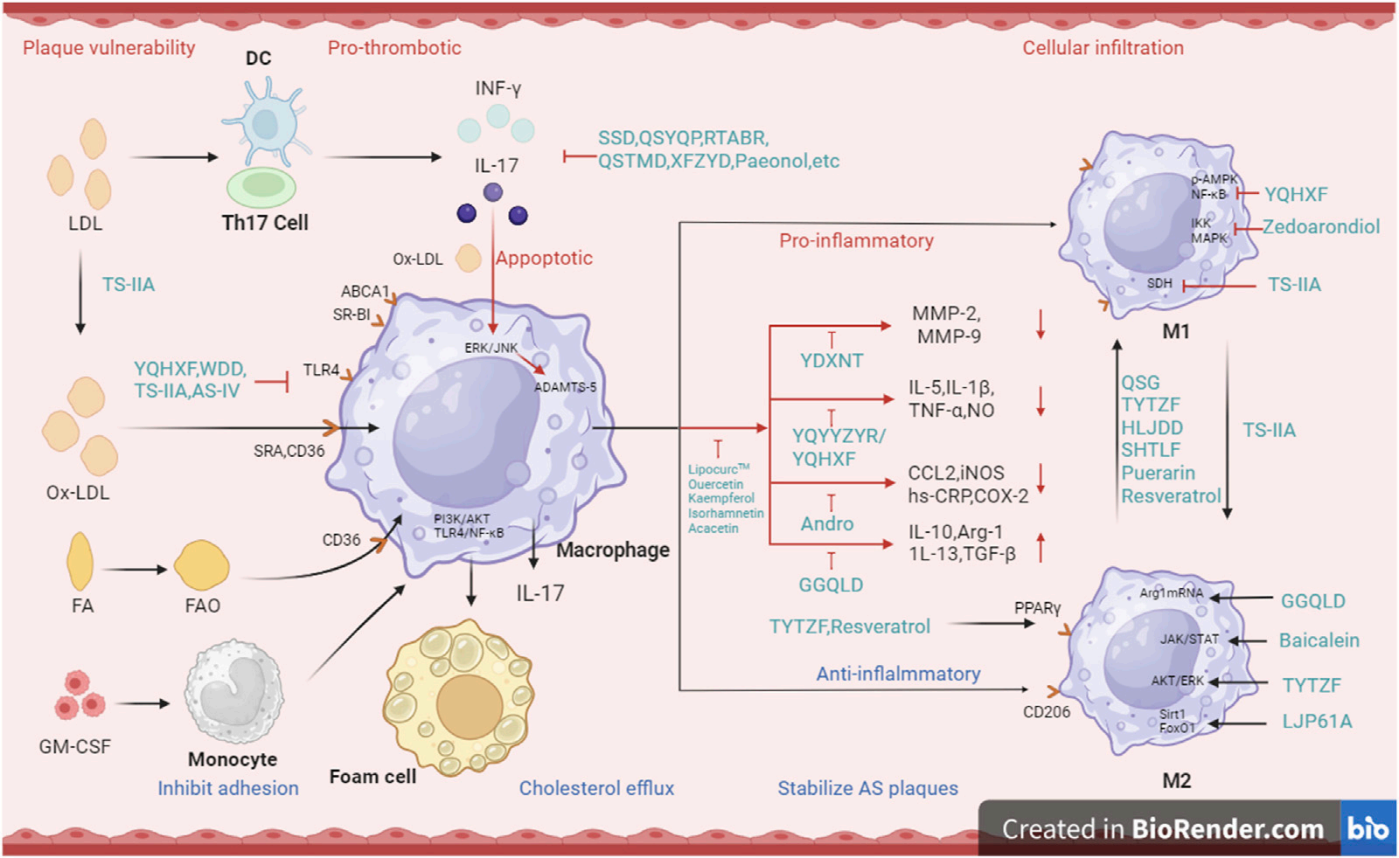
Mechanism of macrophage and IL17 immune microenvironment regulated by Chinese medicine..

In conclusion, macrophages remove lipid deposits, form foam cells, promote plaque formation, trigger inflammation, secrete cytokines, and participate in cell signaling. Ultimately, they contribute to plaque rupture and the development of CHD. Macrophages are crucial in the pathological process of CHD and are important targets for prevention and treatment (Figure 1).

### 2.2 Therapeutic strategies to CHD by modulating macrophages with CM

CM has been shown to modulate the therapeutic effects of macrophages in CHD in both *in vivo* and *in vitro*. For *in vitro* studies, the sources of macrophages mainly include primary cells derived from animal spleen, peritoneum, bone marrow, or monocyte/macrophages lines such as RAW264.7, THP-1, ANA (Figure 1).

#### 2.2.1 CM modulates macrophages by inhibiting inflammation

Studies have shown that CM compounds and their active ingredients have anti-inflammatory effects and can be used to treat CHD by clearing heat, activating blood circulation, resolving blood stasis, and reducing levels of inflammatory factors. Yindan Xinnaotong (YDXNT) inhibits matrix metalloproteinase (MMP)-2/9 expression in macrophages, suggesting its anti-inflammatory effect in controlling CHD (Zhang, 2013). Whereafter, Cheng et al. conducted computational predictions based on network pharmacology and discovered that YDXNT exhibits synergistic anti-atherosclerotic properties by protecting blood vessels, reducing lipids, and providing anti-inflammatory and antioxidant effects. These findings were validated in atherosclerotic rats (Cheng et al., 2015). In patients with restenosis after percutaneous coronary intervention (PCI) for CHD, Ningxin Ditan Decoction (NXDTD) significantly reduced intracellular TC, FC, IL-6, IL-1β, TNF-α levels and the proportion of CD11b^+^CD86^+^ macrophages and apoptosis. It also regulates p-AMPK and nuclear factor kappa-B (NF-κB) signaling to inhibit macrophage differentiation and prevent restenosis (Lijuan et al., 2023). Yiqi Huoxue Formula (YQHXF) (Ningxintong Granule) benefits *Qi* and *Blood*, resolves blood stasis, and relieves pain. YQHXF effectively inhibits foam cell formation by reducing SRA and CD36 protein expression in THP-1-derived macrophages, thus regulating AS (Jisha and Ning, 2015). YQHXF stabilizes AS plaques by affecting phagocytosis of LDL through the toll-like receptor (TLR)-4 pathway in macrophages. Tanyu Tongzhi Formula (TYTZF) alleviates CHD symptoms patients by increasing peroxisome proliferator-activated receptor (PPAR)-γ expression or activating AKT/ERK signaling pathway (Ma et al., 2021). Studies on the pharmacological mechanism of the Tongmai Yangxin Pill (TMYXP), a patented CM, revealed that TMYXP attenuates foam cell formation and lipid deposits by regulating ESR1 and NF-κB signaling pathways, improving CHD patients' biochemical indices (Fan et al., 2021) (Table 1). CM inhibits macrophage activity, reduces inflammatory factor levels, and suppresses their inflammatory response by downregulating NF-κB expression. Network pharmacology is an analytical approach that combines biological networks with pharmacological studies, embodying the holistic philosophy of CM (Ding et al., 2019). Wang et al. identified several molecular targets and pathways associated with *Astragalus membranaceus* and *Angelica sinensis* (A&A) in the treatment of AS. A&A exhibits potential therapeutic effects against AS primarily through anti-inflammatory mechanisms (Wang et al., 2022c). By employing a combination of network pharmacology and experimental validation, the study systematically explored the mechanisms by which Yiqi Huoxue Huatan Recipe (YHHR) treats CAD. It was found that the expression of NF-κB p65 was significantly higher in the high-concentration YHHR group, indicating that YHHR has been shown to combat inflammation and AS through the SRC/NF-κB signaling pathway (Huang et al., 2023). Zedoarondiol exerts anti-inflammatory effects in lipopolysaccharide (LPS)-stimulated macrophages by inhibiting the expression of iNOS, COX-2, and pro-inflammatory cytokines through the inhibition of IKK and MAPK phosphorylation and subsequent inactivation of the NF-κB pathway (Cho et al., 2009). Lipocurc™, a Liposomal Curcumin preparation, effectively reduces pro-inflammatory cytokine/chemokine expression in fibroblasts and macrophages.Compounds in Hawthorn leaves (Quercetin, Kaempferol, and Isorhamnetin) reduced the protein expression of PTGS2, MMP-2, MMP-9, IL-6, IL-1β, TNF-α, and inhibited macrophages activation (Ding et al., 2022) (Table 2).

**TABLE 1:** Therapeutic strategies to CHD by modulating macrophages with CM formulas.

Title	Subjects	Target/Pathway	Mechanism	Reference
Yindan Xinnaotong(YDXNT)	Cell		Inhibit MMP secretion by macrophages	Zhang (2013)
Ningxin Ditan Decoction (NXDTD)	Cell	AMPK/NF-κB pathway	Down-regulate inflammatory factor levels and inhibit macrophages differentiation	Cao et al. (2023)
Yiqi Huoxue Formula (YQHXF) (Ningxintong Granule)	Cell	The “LDL-TLR4-macrophages” pathway	Affect macrophages phagocytosis	Chen et al. (2016)
Yiqi Huoxue Formula (YQHXF) (Ningxintong Granule)	Rats	SRA, CD3	Inhibit foam cell formation	Du and Gu (2015)
Yiqi Huoxue Formula (YQHXF) (Ningxintong Granule)	Cell	SRA, CD3	Inhibit foam cell formation	Du and Gu et al. (2015)
Tanyu Tongzhi Formula (TYTZF)	Mice, Cell	PPARγ and AKT/ERK pathways	Down-regulate the level of inflammatory factors and regulate the transformation of macrophages from M1 to M2	Ma et al. (2021)
Tongmai Yangxin Pill (TMYXP)	Cell	ESR1, NF-κB pathway	Inhibit foam cell formation	Fan et al. (2021)
Yiqi Yangyin Zhuyu Recipe (YQYYZYR)	Cell		Inhibit the polarization of macrophages to the M1 subtype to play an immunomodulatory role	Tang (2019)
Qishen Granule (QSG)	Mice, Cell	AngII/AT1-MCP1/CCL2/CCR2, TGF-β1/Smad3 pathway	Inhibit the release of monocytes from the spleen amd the migration of monocytes to the heart; regulate macrophages subsets; promote angiogenesis	Lu et al. (2019)
Gegen Qinlian Decoction (GGQLD)	Mice		Reduce blood lipid levels and regulates the transformation of macrophages from M1 to M2	Zheng et al. (2022)
Tanyu Tongzhi Formula (TYTZF)	Mice, Cell	PPARγ, AKT/ERK pathway	Down-regulate the level of inflammatory factors and regulate the transformation of macrophages from M1 to M2	Ma et al. (2021)
Huanglian Jiedu Decoction (HLJDD)	Mice, Cell	AMPK	Down-regulate the level of inflammatory factors and regulate the transformation of macrophages from M1 to M2	Li et al. (2021)
Shenhong Tongluo Formula (SHTLF)	Mice		Regulate the transformation of macrophages from M1 to M2	Shi et al. (2021)
Wendan Decoction (WDD)	Cell		Reduce endocytosis and efflux of cholesterol and resist macrophages foaminess	Chen et al. (2019)
Qingxin Jieyu Formula (QXJYF)			(Web Pharmacology Screening) Macrophages iron death stabilizes AS-vulnerable plaques	Lai et al. (2021)
Yiqi Huoxue Decoction (YQHXD)	Patients		Improve the response to inflammatory factors	Gao et al. (2012)

**TABLE 2:** Therapeutic strategies to CHD by modulating macrophages with CM compounds.

Title	Subjects	Target/Pathway	Mechanism	References
Zedoarondiol	Cell	IKK/MAPK/NF-κB pathway	Inhibit iNOS, COX-2 and pro-inflammatory cytokines	Cho et al. (2009)
Liposomal Curcumin Formulation (Lipocurc™)	Cell		Downregulate the level of inflammatory factors	
Quercetin, Kaempferol and Isorhamnetin	Cell		(Web Pharmacology Screening) Reduce the protein expression of PTGS2, MMP-2, MMP-9, IL-6, IL-1B, TNF-α, and inhibite macrophages activation	Ding et al. (2022)
Tanshinone IIA (TS-IIA)	Cell	SIRT1 pathway	Regulate the transformation of macrophages from M1 to M2	Song (2022)
Tanshinone IIA (TS-IIA)	Mice	miR-375/KLF4 pathway	Reduce inflammatory factors and blood lipid levels, improve autophagy and M2 polarization of macrophages	Chen et al. (2019)
Baicalin	Rats	JAK/STAT pathway	Downregulate the level of inflammatory factors and regulate macrophages subsets	Xu et al. (2020)
Baicalin	Cell	METRK receptor	Promote macrophages burial and anti-inflammatory activity, mediate M2 macrophages polarization	Lai et al. (2018)
Laminaria japonica polysaccharide (LJP61A)	Mice, Cell	SIRT1, FoxO2	Inhibit foam cell formation and regulate macrophages polarization and autophagy	Li et al. (2022a)
Acacetin			Inhibit M1 polarization and suppress pro-inflammatory cytokine expression	Ouyang et al. (2021)
Andrographolide	Rats		Downregulate the level of inflammatory factors and regulate macrophages subsets	Shu et al. (2020)
Puerarin	Mice	SIRT3 pathway	Inhibit macrophages apoptosis and reduce macrophages infiltration	Li et al. (2007)
Resveratrol	Cell	PPA-γ pathway	Inhibits MMP secretion by macrophages	Ge et al. (2006)
Formononetin	Mice		Change the plaque composition and weaken the formation of VSMC and macrophages-derived foam cells and their accumulation on the arterial wall to enhance the stability of the lesion	Ma et al. (2021)
Flavonoids	Cell		Promote macrophages cholesterol efflux, resist macrophages foaming, inhibit the secretion of inflammatory factors, and induce macrophages apoptosis induced by antioxidant modified ox-LDL	Li et al. (2020)
Ginger (Zingiber officinale Roscoe)			Promotes cholesterol efflux from macrophages	Li (2021a)
Salviain	Cell		Promotes cholesterol efflux from macrophages	Qian et al. (2012)
TS-IIA, AS-IV	Mice, Cell	PI3K/AKT, TLR4/NF-κB pathway	Play the role of anti-inflammation and antioxidation to regulate plaque stability	Wang et al. (2022d)
TS-IIA	Mice, Cell	SDH	Suppress pro-inflammatory cytokine expression	Liu et al. (2021)
TS-IIA			(Review Screening) Inhibit LDL oxidation, monocyte adhesion to endothelium, macrophages cholesterol accumulation, pro-inflammatory cytokine expression, and platelet aggregation	Lu (2021)

The above data suggest that CM formulas such as YDXNT, NXDTD, YQHXF, TYTZF, TMYXP, YHHR (Table 5), CM ingredients such as Zedoarondiol, and the novel nanoparticle Lipocurc™, can reduce macrophage inflammation. These *in vitro* studies indicate NF-κB, AMPK, PI3K/AKT, and chemokine (motif C-C) ligand (CCL)-2/CC chemokine receptor (CCR)-2, may become key targets for the regulation of macrophages by CM in the treatment of CHD.

#### 2.2.2 CM modulates macrophages by regulating their polarization

CM can regulate macrophage subpopulations and decrease levels of inflammatory factors. *In vitro* experiment, Yiqi Yangyin Zhuyu Recipe (YQYYZYR) could reduce the secretion of inflammatory factors such as NO, TNF-α, and IL-6 by inhibiting M1 macrophage polarization (Tang, 2019). Gegen Qinlian Decoction (GGQLD) increases the expression of Arg-1 and CD206 while decreasing iNOS expression, indicating its ability to regulate lipid metabolism and macrophage polarization, improving the inflammatory microenvironment (Yi, 2022). In addition, Qishen Granule (QSG) (Lu et al., 2019), TYTZF (Ma et al., 2021), Huanglian Jiedu Decoction (HLJDD) (Biren, 2021; Li, 2021a; Li, 2021b), and Shenhong Tongluo Formula (SHTLF) (Yanyu, 2022) have been found to regulate macrophage subpopulations and switch M1 to M2 macrophages, reducing inflammation and the development of AS. Tanshinone IIA (TS-IIA) has been shown to activate the Sirt1 pathway and convert macrophages from M1 to M2 type, stabilizing AS plaques (Song, 2022; Chen et al., 2019). Baicalin from *Scutellaria baicalensis* (Yin-Siew Lai) has a protective effect on the heart by altering macrophage polarization and reducing inflammation. It decreases iNOS, IL-1β, and IL-6 levels while increasing Arg-1, IL-10, and TGF-β levels (Lai et al., 2018). Baicalin also attenuates myocardial injury after I/R through the JAK/STAT pathway (Xu et al., 2020). Furthermore, Laminaria japonica polysaccharide (LJP61A) enhances autophagic flux of macrophages by upregulating the key upstream genes of autophagy, Sirt1 and FoxO1 and alleviating inflammatory responses in AS (Li X. Y. et al., 2022). Acacetin from *Sparganii rhizoma* (Yuanshuo Ouyang) attenuates LPS-induced M1 polarization and pro-inflammatory cytokine expression, but its specific mechanism and pathway are still unknown (Ouyang et al., 2021). Andrographolide, an active compound extracted from *Andrographis paniculata* (Jia Shu), decreases TNF-α, MCP-1, high-sensitivity C-reactive protein (hs-CRP), and IL-1β levels by altering macrophage phenotype in mice (Shu et al., 2020). Additionally, Puerarin and Resveratrol (Ge et al., 2006), active ingredients of CM, regulate macrophage polarization, with Resveratrol agonizing PPARγ to inhibit the expression of extracellular MMP inducers.

The aforementioned data demonstrate that CM formulas, including YQYYZYR, QSG, GGQLD, TYTZF, SHTLF (Table 5), and active ingredients such as Baicalin, TS-IIA, Laminaria japonica polysaccharide (LJP61A), Acacetin, Andrographolide, Puerarin, and Resveratrol, are effective in regulating macrophages polarization and reducing the intensity of inflammatory response. Thus, these CM mitigate the aggravation of AS lesions and protect the heart (Table 1).

#### 2.2.3 CM modulates macrophages by promoting cholesterol efflux

CM was found to be effective in promoting cholesterol efflux from macrophages and inhibiting foam cell formation. They can promote the phagocytosis and clearance of LDL by macrophages, reducing the deposition of LDL in the vessel wall and mitigating the development of CHD. It’s found that Wendan Decoction (WDD) may reduce cholesterol internalization and phagocytosis by macrophages by inhibiting the expression of macrophages membrane protein CD36 and SRA. Simultaneously, it upregulates the protein expression of macrophages membrane protein ATP-binding cassette transporter A1 (ABCA1) and class B type I scavenger receptor (SR-BI), promoting cholesterol efflux from macrophages (Xuanjing, 2020). Based on Network pharmacology research, Runmin Lai analyzed the important active ingredients in Qingxin Jieyu Formula (QXJYF) and found that 110 active ingredients of five CM (i.e., Salvia miltiorrhiza, persimmon) in the formula could act on 87 target genes. Macrophages iron death regulation might be its main potential pathway to stabilize AS-prone plaques (Runmin, 2021) (Table 1). Formononetin, an isoflavone from *Astragalus membranes* (Chuanrui Ma), improves lesion stability, attenuates foam cell formation, and reduces plaque accumulation. It also inhibits monocyte adhesion and inflammation, delaying AS onset (Ma et al., 2020). Flavonoids, the active ingredients of CM, have significant cardiovascular effects. Some flavonoids can act on macrophages through different pathways and mechanisms to exert different degrees of anti-AS effects. The related genes are ABCA1, ATP-binding cassette protein G1, TLR, and SRA (Li et al., 2020). *Ginger* (Li, C.) components alleviate oxidative stress, inflammation, and promote NO synthesis, and enhance cholesterol efflux, inhibit angiogenesis, and induce autophagy. Clinical trials show benefits for blood lipids, inflammatory cytokines, blood pressure, and platelet aggregation (Li C. et al., 2021). Ginger and its components have potential in treating hypertension, coronary artery disease, peripheral artery disease, and other vascular conditions. Macrophages upregulate liver X receptor (LXR) and its target genes in response to Salviain, enhancing cholesterol efflux and exerting myocardial protective effects (Qian et al., 2012). TS-IIA and Astragaloside IV (AS-IV) are natural CM extracts that have cardioprotective effects by reducing inflammation and oxidative stress in AS. They enhance plaque stability through PI3K/AKT and TLR4/NF-κB signaling and inhibit cytoplasmic lipid droplet accumulation in macrophages (Wang et al., 2020). TS-IIA also prevents LPS-induced inflammation by targeting succinate dehydrogenase (SDH) in macrophages (Liu et al., 2021). Additionally, TS-IIA inhibits LDL oxidation, monocyte adhesion, macrophage cholesterol accumulation, pro-inflammatory cytokine expression, and platelet aggregation, making it a potential treatment for stabilizing AS plaques (Gao et al., 2012) (Table 2).

Based on the above studies, we concluded that CM formulas such as WDD, YQHXF, QXJYF (Table 5), and CM components such as Formononetin, Flavonoids, Ginger components, Salviain, Tea polyphenols, TS-IIA, and AS-IV can inhibit foam cell formation or regulate ferroptosis in macrophages by reducing lipid deposition in macrophages, stabilizing vulnerable plaques and resisting the formation of AS.

## 3 CM modulates the pathogenic role of IL-17 in CHD development

### 3.1 Mechanism of IL-17 in CHD progression

Leukocytes in the arterial wall contribute to AS plaques formation. IL-17, produced by leukocytes like T helper (Th)17 cells, is an inflammatory cytokine that plays a crucial role in host defense. Elevated IL-17A levels are associated with cardiovascular disease and are a key factor in AS development. IL-17 triggers cytokine cascades, accelerating AS and plaque vulnerability. A study found that IL-17 levels decreased significantly after treatment in angina patients, indicating its involvement in the inflammatory response and clinical instability in CHD (Boluri et al., 2022). macrophages cell line THP-1 was treated with 100 ng/mL of IL-17A for 24 h and the mRNA and protein expression levels of ADAMTS-5 proteases were significantly upregulated and peaked at 8 h. Subsequent inhibition studies showed that IL-17A upregulation of ADAMTS-5 was mediated through the ERK and JNK pathways in THP-1 cells (Thou et al., 2020). Studies showed IL-17A induces pro-inflammatory, pro-thrombotic responses, increases plaque vulnerability, and attracts cells in human plaque tissue samples (Akhavanpoor et al., 2017). During atherogenesis, the IL-17A/IL-17RA axis increases aortic arch inflammation by inducing aortic chemokines and accelerates neutrophil and monocyte recruitment to this site (Butcher et al., 2012).

In addition, IL-17A induced a mixed macrophage-DC phenotype, previously identified in the context of AS as a major regulator of cholesterol homeostasis in macrophages. IL-17A has a monocyte/macrophages profile with a significant effect on the monocyte/macrophages profile in atherogenesis (Salvatore et al., 2015). Inhibiting IL-17A could reduce inflammatory load, cellular infiltration, and improve plaque stability, thus preventing AS progression (Erbel et al., 2014) (Figure 1).

### 3.2 Therapeutic strategies to CHD by modulating IL-17 with CM

CM compounds have anti-inflammatory effects, reducing IL-17 levels. Paeonol, extracted from *Paeonia suffruticosa* (Xiaoyan Shi), restores Treg/Th17 balance by modulating gut microbiota. This reduces pro-inflammatory cytokines and MMP expression in aortic inflammatory cells. Paeonol also increases anti-inflammatory cytokine IL-10, indicating its effectiveness in anti-AS (Shi et al., 2021). Cycloastragenol (Y006), a natural product, has potent anti-inflammatory properties and protects myocardial cells. *In vivo* and *in vitro* studies show Y006 inhibits TNF-α, IFN-γ, and IL-17 production during hypoxia and myocardial infarction. It also increases IL-10 and IL-4 expression, enhancing myocardial function by regulating molecular genes under hypoxia and apoptosis (Ren et al., 2020) (Table 3).

**TABLE 3:** Therapeutic strategies to CHD by modulating IL-17 with CM compounds.

Title	Subjects	Target/Pathway	Mechanism	References
Paeonol	Rats		Suppress pro-inflammatory cytokine expression	Shi et al. (2021)
Cycloastragenol (Y006)	Mice		Suppress pro-inflammatory cytokine expression	Ren et al. (2020)
Quercetin		MAPK, PI3K/AKT, IL-17 pathway, etc	Web Pharmacology Screening	Wu et al. (2019)
Ficus hirta-Hypericum perforatum		PI3K/AKT, IL-17, HIF-1 pathway, etc	Web Pharmacology Screening	Lai et al. (2021)

Furthermore, CM formulation play a crucial role in reducing IL-17 inflammatory cytokine levels and improving the treatment of CHD. In an animal study, Shenshao Decoction (SSD) decreased TC, TG, and LDL-C levels, with the high-dose group showing a significant reduction in IL-1β, IL-17A and IL-23. This suggests SSD suppresses the inflammatory response and attenuates plaque progression (Xue et al., 2013). Qishen Yiqi Pill (QSYQP) increased CD36 expression, reduced IL-17A generation, decreased plaque formation and LDL levels. It achieves anti-atherogenic effects through mechanisms like promoting regulatory T cells, facilitating hepatic cholesterol excretion, and inhibiting plaque formation and Th17 cells (Peng et al., 2017). Furthermore, Yiqi Huoxue Decoction (YQHXD) improved symptoms and heart function in *Qi* deficiency and blood stasis patients after PCI. IL-17 and hs-CRP levels significantly decreased after treatment, indicating improved inflammatory factor response and heart function (Lu, 2021) (Table 4).

**TABLE 4:** Therapeutic strategies to CHD by modulating IL-17 with CM formulas.

Title	Subjects	Target/Pathway	Mechanism	Reference
Shenshao Decoction(SSD)	Rats		Inhibit inflammatory response and reduce lipid levels	Xue et al. (2013)
QishenYiqi Pill (QSYQP)	Mice		Promote regulatory T cells and hepatic cholesterol excretion, plaque suppression and Th17 cells	Peng et al. (2017)
Buyang Huanwu Decoction (BYHWD)	Rats		Reduce the expression of inflammatory factors and affect the IL-17 signaling pathway	Wang et al. (2022a)
Yiqi Huoxue Decoction (YQHXD)			Improve inflammatory factors	Gao et al. (2021)
Removing Toxins to Activate Blood Recipe (RTABR)			Web Pharmacology Screening	Liu et al. (2021)
Yiqi Ningshen Tablet (YQNST)		MAPK, PI3K/AKT, IL-17 pathway etc	Web Pharmacology Screening	Lv et al. (2020)
Qishao Tongmai Decotionon (QSTMD)			Web Pharmacology Screening	Chunlin (2020)
Xuefu Zhuyu Decoction (XFZYD)			Web Pharmacology Screening	Yang et al. (2023)

Network pharmacological analysis suggests that CM formulas and compounds can treat CHD through the IL-17 pathway. Quercetin, a flavonoid extracted from *Ginkgo biloba leaves* (Xiaojun Wu), has potential therapeutic effects in the treatment of cardiovascular diseases. Network pharmacology analysis reveals that quercetin regulates MAPK, IL-17, and phosphatidylinositol-3-kinase/protein kinase B (PI3K/AKT) signaling pathways for cardiovascular disease treatment (Wu et al., 2019). Ficus hirta-Hypericum perforatum components and targets alleviate microvascular angina through PI3K/AKT, IL-17, and HIF-1 signaling pathways, reducing oxidative stress and inflammation (Lai et al., 2021). Buyang Huanwu Decoction (BYHWD) decreases myocardial fibrosis and inhibits plaque formation by modulating the IL-17 pathway and reducing inflammatory factors (Wang et al., 2022d). Yiqi Ningshen Tablet, containing multiple active ingredients, is commonly used in CM for CHD treatment. It modulates CHD through the IL-17 pathway (Lv et al., 2020).

Common CM formulas, such as Removing Toxins to Activate Blood Recipe (RTABR) (Suli, 2021), Qishao Tongmai Decoction (QSTMD) (Chunlin, 2020), and Xuefu Zhuyu Decoction (XFZYD) have been widely used (Table 5). However, the mechanism of action of these CM formulas still requires further exploration by researchers (Figure 1).

**TABLE 5:** CM compound and its composition.

Prescription	Ingredient	References
Yindan Xinnaotong (YDXNT)	*Ginkgo biloba leaf, Salvia miltiorrhiza (or Danshen), Rhizoma Asari (or Asarum), Gynostemma pentaphyllum (or Jiao Gu Lan), Hawthorn (or Crataegus), Garlic (or Allium sativum), Panax notoginseng (or Notoginseng), Mugwort slices (or Ai Ye)*	Zhang (2013)
Ningxin Ditan Decoction (NXDTD)	*Peach kernel (or Prunus persica), Safflower (or Carthamus tinctorius), Pinellia tuber (or Banxia), Bamboo shavings (or Zhuru), Trifoliate orange fruit (or Zhi Shi), Poria (or Poria cocos), Aged tangerine peel (or Chen Pi), Fresh ginger (or Zingiber officinale), Licorice (or Glycyrrhiza uralensis)*	Lijuan et al. (2023)
Yiqi Huoxue Formula (YQHXF) (Ningxintong Granule)	*Ginseng, Poria, Angelica Sinensis, Chuanxiong Rhizoma, Sour Jujube Seed, Licorice*	Jisha and Ning (2015)
Tanyu Tongzhi Formula (TYTZF)	*Cucurbitaceae/Y.-J.Jin1 (Quan Gua Lou), Lamiaceae/Y.-J.Jin 2 (Dan Shen), Amaryllidaceae/Y.-J.Jin 3 (Xie Bai), Hirudinidae/Y.-J.Jin 4 (Shui Zhi), Araceae/Y.-J.Jin 5 (Shi Chang Pu), Zingiberaceae/Y.-J.Jin 6 (Yu Jin), Polyporaceae/Y.-J.Jin 7Rutaceae/Y.-J.Jin 8 (Fu LingChen Pi)*	Ma et al. (2021)
Tongmai Yangxin Pill (TMYXP)	*Rehmannia glutinosa (Gaertn.) DC., Spatholobus suberectus Dunn, Ophiopogon japonicus (Thunb.) Ker Gawl., Glycyrrhiza uralensis Fisch., Polygonum multiflorum Thunb., Equus asinus L., Schisandra chinensis (Turcz.) Baill., Codonopsis pilosula (Franch.) Nannf., Chinemys reevesii (Gray), Ziziphus jujuba Mill. and Cinnamomum cassia (L.) J.Presl (Committee of the Pharmacopoeia of PR China, 2015)*	Fan et al. (2021)
Yiqi Huoxue Huatan Recipe (YHHR)	*Main ingredients: Astragalus membranaceus, Salvia miltiorrhiza, Hirudo, Coptis chinensis*	Huang et al. (2023)
Gegen Qinlian Decoction (GGQLD)	*Kudzu Root (Ge Gen), Scutellaria Baicalensis (Huang Qin), Coptis Chinensis (Huang Lian), Licorice Root (Gan Cao)*	Yi (2022)
Qishen Granule (QSG)	*A. membranaceus (Fisch.)Bunge., S. Miltiorrhiza Bunge., L. japonica Thunb., S. aestivalis Griseb., A. fischeri Rchb. 和 G. uralensis Fisch*	Lu et al. (2019)
Huanglian Jiedu Decoction (HLJDD)	*Coptis Chinensis (Huang Lian), Scutellaria Baicalensis (Huang Qin), Phellodendron Amurense (Huang Bai), Gardenia Fruit (Zhi Zi)*	Biren (2021), Li (2021a), Li (2021b)
Shenhong Tongluo Formula (SHTLF)	*Ginseng (Ren Shen), Salvia Miltiorrhiza (Dan Shen), Rhodiola Rosea (Hong Jing Tian), Honeysuckle (Jin Yin Hua), Red Peony Root (Chi Shao), Trichosanthes Kirilowii (Gua Lou), Angelica Sinensis (Dang Gui), Sandalwood (Jiang Xiang)*	Yanyu (2022)
Wendan Decoction (WDD)	*Pinellia Ternata (Ban Xia), Bamboo Shavings (Zhu Ru), Bitter Orange (Zhi Shi), Orange Peel (Ju Pi), Licorice Root (Gan Cao), Fresh Ginger (Sheng Jiang)*	Xuanjing (2020)
Qingxin Jieyu Formula (QXJYF)	*Astragalus Root (Huang Qi), Salvia Miltiorrhiza (Dan Shen), Ligusticum Wallichii (Chuan Xiong), Patchouli (Huo Xiang), Coptis Chinensis (Huang Lian)*	Runmin (2021)
Shenshao Decoction (SSD)	*Radix Angelicae Sinensis (Ren Shen), Ligusticum wallichii (Chuan Xiong), White Peony Root (Bai Shao), Aconite (Fu Zi), Poria (Fu Ling), Honey-Fried Licorice (Zhi Gan Cao), Schisandra (Wu Wei Zi)*	Xue et al. (2013)
Qishen Yiqi Pill (QSYQP)	*Radix et Rhizoma Salviae Miltiorrhizae (Dan Shen), Radix Astragali (Huang Qi), Radix et Rhizoma Notoginseng (San Qi), Lignum Dalbergiae Odoriferae (Jiang Xiang) (Jing Wang)*	Peng et al. (2017)
Yiqi Huoxue Decoction (YQHXD)	*Codonopsis Pilosula (Dang Shen), Panax Notoginseng (San Qi), Astragalus (Huang Qi), Salvia Miltiorrhiza (Dan Shen), Ligusticum Wallichii (Chuan Xiong), Rhodiola Rosea (Hong Jing Tian), Corydalis Yanhusuo (Yan Hu Suo), Bupleurum (Chai Hu), Ophiopogon Japonicus (Mai Dong), Sour Jujube Seed (Suan Zao Ren), Schisandra (Wu Wei Zi), Dalbergia Wood (Xiang), Amomum (Sha Ren)*	Lu (2021)
Buyang Huanwu Decoction (BYHWD)	*Astragalus membranaceus (Huang Qi), Angelica sinensis (Dang Gui), Radix Paeoniae Rubra (Chi Shao), Lumbricus (Di Long), Ligusticum chuanxiong (Chuan Xiong), Carthamus tinctorius (Hong Hua), and Semen Persicae (Tao Ren)*	Wang et al. (2022d)
Removing Toxins to Activate Blood Recipe (RTABR)	*Scutellaria Baicalensis (Huang Qin), Coptis Chinensis (Huang Lian), Salvia Miltiorrhiza (Dan Shen), Red Peony Root (Chi Shao), Ligusticum Wallichii (Chuan Xiong) (Source: First Affiliated Hospital of Guangzhou University of Chinese Medicine, Cardiology Department)*	Suli (2021)
Qishao Tongmai Decoction (QSTMD)	*Astragalus (Huang Qi), Red Peony Root (Chi Shao), Ligusticum Wallichii (Chuan Xiong), Dalbergia Wood (Jiang Xiang), Pueraria (Ge Gen), Sour Jujube Seed (Suan Zao Ren)*	Chunlin (2020)
Xuefu Zhuyu Decoction (XFZYD)	*Angelica sinensis (Oliv.) Diels, Rehmannia glutinosa (Gaertn.) DC., Prunus persica (L.) Batsch, Carthamus tinctorius L., Paeonia lactiflora Pall., Citrus × aurantium L., Glycyrrhiza glabra L., Bupleurum chinense DC., Ligusticum striatum DC., Platycodon chinensis Lindl. and Paxton and Achyranthes bidentata Blume*	

## 4 Effects of IL-17-regulated macrophages in CHD development

### 4.1 Mechanism of IL-17-regulated macrophages in CHD

IL-17 has been extensively studied in various diseases, including CHD. It is mainly produced by Th17 cells, γδ T cells, and CD8^+^ T cells (Ghoreschi et al., 2011). IL-17 indirectly affects macrophage polarization. Studies have shown that IL-17 treatment induces a unique transcriptional pattern in human monocyte-derived macrophages, different from other macrophage phenotypes (Erbel et al., 2014). This finding is supported by another study that found no increase in the expression of M1 or M2-associated surface molecules with IL-17 (de la Paz Sanchez-Martinez et al., 2017).

IL-17-regulated macrophages contribute to AS in CHD. Macrophages are crucial for activating T cells and initiating immune responses (Guerriero, 2019). The balance of M1 and M2 phenotypes in plaque macrophages can be influenced by the pro-inflammatory or anti-inflammatory T cell phenotype (de la Paz Sanchez-Martinez et al., 2017). The complex interactions between macrophages and T cells can be targeted for CHD treatment (Song et al., 2019). T lymphocytes are abundant and activated in AS plaques. Th1 secretes IFN-γ, IL-2, and TNF-α, while Th17 cells secrete IL-17, which activates macrophages and promotes inflammation. IL-17-producing T cells, expressing IFN-γ, are highly expressed in AS lesions (Lu, 2017). Elevated levels of IL-17 have been found in AS patients, indicating its involvement in AS development (de Boer et al., 2010). However, IL-17E (also named IL-25) produced by macrophages may have a protective effect on AS. It regulates macrophages, promotes lipolysis, inhibits lipogenesis, and enhances mitochondrial respiratory capacity (Feng et al., 2018).

IL-17A has a pro-atherogenic effect by inducing aortic macrophages, Th1-related cytokine, and aortic chemokine expression (Butcher and Galkina, 2011). It increases plaque fragility and promotes macrophage apoptosis without affecting phagocytosis of apoptotic cells (Wang B. et al., 2022). IL-17A also upregulate the expression of certain chemokines in plaques, further weakening arterial wall plaques. Another study (Wang, 2014) showed that IL-17A intervention increased plaque lipid content, necrotic core volume, T cells infiltration, and apoptosis, while reducing fibrous cap thickness and plaque stability. It also increases macrophage apoptosis and chemokine content, without affecting macrophage apoptotic clearance. Abnormal lipid metabolism plays a significant role in the interaction between IL-17 and macrophages in CHD. A systematic analysis revealed that increased IL-17A expression leads to elevated levels of cytokines such as IL-6 and TGF-β in experimental AS and clinical data. IL-17A also promotes infiltration of aortic macrophages and T cells in most AS models. Additionally, inhibition of TANK (TRAF family member associated NF-κB activator)-binding kinase 1 (TBK1) reduces IL-17-induced inflammation (Lee et al., 2017). In patients with diabetes and CHD, hyperglycemia activates macrophages via TBK1-HIF-1α-mediated IL-17/IL-10 signaling, contributing to coronary artery AS complexity (Li Q. et al., 2021).

Additionally, IL-17A may also exert anti-inflammatory effects by regulating aortic vascular cell adhesion molecule (VCAM)-1 expression and T cells content (Butcher and Galkina, 2011). Human adipose tissue-derived mesenchymal stromal cells (AT-MSCs) can polarize macrophages to an anti-inflammatory phenotype, reducing the secretion of inflammatory cytokines and macrophage phagocytosis (Adutler-Lieber et al., 2013). These studies suggest there may also be a beneficial aspect for IL-17-related macrophages in CHD. Anyway, IL-17 holds potential as a therapeutic target for cardiovascular diseases.

### 4.2 Therapeutic research on the modulation of IL-17-regulated macrophages by CM

IL-17 signaling pathway plays a role in macrophage differentiation and susceptibility to AS in systemic lupus erythematosus (SLE) (Wang et al., 2022f). Animal experiments on the active ingredients of CM have revealed that Formononetin inhibited M1 polarization and promoted M2 polarization in macrophages/microglia, thereby improving cardiac function in mice with myocardial infarction and depression by reversing polarization. Importantly, IL-6 and IL-17A produced post-myocardial infarction may cause neuroinflammation, and mancostemonin attenuates this process (Yang et al., 2023). Kedaling tablets (KDL), a Chinese patented medicine originated from *Corydalis yanhusuo* (Y.H. Chou & Chun C.Hsu), are prescribed for the prevention of AS. Kedaling tablets can attenuate atherosclerotic plaque and reduce IL-1β and IL-17 in ApoE−/− mice. The anti-atherosclerotic effects of Kedaling tablets may be associated with the suppression of inflammatory signaling pathways (Li et al., 2024).

CM has shown potential in modifying IL-17-regulated macrophages in CHD, as seen in other inflammatory diseases. Dihydrosanguinarine (DS) is a chemical component found in Corydalis bungeana Turcz. It has been found to have *in vitro* anti-inflammatory, antioxidant, and antimicrobial properties. Network pharmacology methods have predicted DS targets for anti-inflammatory effects, including AKT3, PI3KCA, CCL2, FOS, IL-17A, IL-17RA, IL-17RE, IL-1β, IL-6 and TNF-α. Additionally, DS has been shown to primarily reduce macrophage infiltration induced by LPS, exerting an anti-hepatitis effect (Xiang et al., 2022). Both *in vitro* and *in vivo* experiments have shown that XFBD can downregulate the secretion of pro-inflammatory cytokines (such as IL-6, TNF-α, IL-1β, and iNOS) by macrophages, and its main component, glycyrrhizic acid, has a strong affinity for IL-17A. This reduces inflammatory reactions, potentially reducing macrophage infiltration and alleviating acute lung injury (Wang et al., 2022g). In intracerebral hemorrhage, hemoglobin induces the formation of TLR2/TLR4 heterodimers in macrophages, leading to increased IL-23 expression. IL-23 stimulates γδ T cells to produce IL-17, exacerbating secondary brain injury. Sparstolonin B (SsnB) in CM can inhibit the formation of TLR2/TLR4 heterodimers, potentially improving this process (Zhong et al., 2016).

IL-17A production can have different effects on macrophages. Hochu-ekki-to (TJ-41), a traditional Japanese herbal medicine, increases IL-17A production, which promotes macrophage production and clearance of pneumococcus (Nakakubo et al., 2020). In the treatment of refractory liver cancer with Oxaliplatin, M2 macrophages overexpress IL-17, reducing the pro-apoptotic effect of Oxaliplatin on tumor cells. Targeting the IL-17 pathway may help suppress liver cancer development (Guo et al., 2017). “Dragon’s blood” is a deep red resin primarily derived from *the dragon blood tree (Dracaena cochinchinensis)*. It is valued in CM for its significant effects on improving blood circulation and dissipating blood stasis. Recent studies have revealed that dragon’s blood shows considerable potential in the treatment of coronary heart disease by promoting blood flow and reducing blood viscosity, thereby improving cardiac blood supply and helping to alleviate chest pain and decrease the frequency of angina attacks (Fan et al., 2014). A comparison of the antiplatelet aggregation effects of two types of “dragon’s blood resin,” Daemonorops draco (RDD) and Dracaena cochinchinensis (RDC), led to the selection of the more potent RDD as the primary component to enhance the efficacy of the formula. In clinical studies, the effects of “Xuefu Zhuyu Decoction” on platelet aggregation and endothelial function in patients with coronary heart disease were observed to evaluate its efficacy and safety (Yi et al., 2011). Additionally, diosgenin, the main active metabolite of *Dioscorea nipponica (DN)* (Jia-Fu Feng), exerts an anti-myocardial ischemia effect by enhancing the body’s antioxidant capacity. In one study, three types of diosgenin (DN, D. panthaica, and D. zingiberensis) significantly reduced oxidative stress markers and inflammatory factors in myocardial tissue, thereby demonstrating an anti-myocardial ischemia effect (Feng et al., 2017; Tang et al., 2015).

The above studies indicate that IL-17 and macrophages are important in cardiovascular diseases. Comparing the treatment of other diseases with CM can help to identify the mechanisms of CM in regulating IL-17-regulated macrophages and their potential application in CHD. This can expand the possibilities for CM in CHD treatment.

## 5 Discussion

Both IL-17 and macrophages play a significant role in the development of CHD. The main dysfunctional macrophages form foam cells, promote plaque formation and inflammation, leading to destabilization of the fibrous cap, plaque rupture and CHD development, although there is also few studies show that IL-17 induces anti-inflammatory macrophages to reduce inflammation, the role of IL-17 remains controversial. Thus, it is crucial to modulate and balance the functions of IL-17 related macrophages during the prevention and treatment of CHD. Macrophages and Th17 cells, which produce IL-17, are important in hypertension, while studies on atherosclerosis have produced conflicting findings regarding the impact of IL-17 and Th17 cells on disease progression and plaque stability. Therefore, further research on IL-17A is warranted.

We performed the Mendelian randomization (MR) analysis between single-nucleotide polymorphisms (SNPs) and other genetic variants of IL-17a and IL-17ra from GWAS focusing on cardiovascular diseases, which was showed in Table 6. There was not enough SNPs passed the genetic variants threshold screening for IL17a. The MR results of IL-17 receptors show that for IL17RA, there is no significant association with CAD, CHD, or CA, as indicated by the non-significant p-values (ranging from 0.5 to 0.95) and odds ratios (ORs) close to 1. For IL17RB, there is a suggestive negative association with CAD and CHD (OR = 0.95, p = 0.069), but no significant association with coronary atherosclerosis. IL17RC shows a significant negative association with CAD (OR = 0.96, p = 2.50E-08) and a stronger significant negative association with coronary atherosclerosis (OR = 0.88, p = 6.00E-09). The inverse variance weighted method was used with 2 or 3 SNPs for each analysis. Although we found statistical significance in the results above, there were conflicts among different casual relationship between IL-17 receptors subsets and the cardiovascular diseases. The interpretation of these results need to be further investigated and discussed in a more rigorously designed MR study.

**TABLE 6:** Mendelian randomization (MR) analysis between IL-17 receptors and cardiovascular diseases from GWAS databases.

ID exposure	ID outcome	Exposure	Outcome	Methods	Nsnp	Beta	SE	P-value	OR
eqtl-a-ENSG00000177663	DGWAS-5533	IL17RA	Coronary artery disease	Inverse variance weighted	2	0.01	0.01	0.5	1.01
eqtl-a-ENSG00000177663	DGWAS-5511	IL17RA	Coronary artery disease	Inverse variance weighted	2	0.04	0.07	0.6	1.04
eqtl-a-ENSG00000177663	DGWAS-5707	IL17RA	Coronary heart disease	Inverse variance weighted	2	0.04	0.07	0.6	1.04
eqtl-a-ENSG00000177663	DGWAS-12	IL17RA	Coronary atherosclerosis	Inverse variance weighted	2	−0.01	0.05	0.85	0.99
eqtl-a-ENSG00000177663	DGWAS-3027	IL17RA	Coronary atherosclerosis	Inverse variance weighted	2	−0.01	0.08	0.95	0.99
eqtl-a-ENSG00000056736	DGWAS-5485	IL17RB	Coronary artery disease	Inverse variance weighted	2	0.02	0	0	1.02
eqtl-a-ENSG00000056736	DGWAS-5511	IL17RB	Coronary artery disease	Inverse variance weighted	3	−0.05	0.03	0.069	0.95
eqtl-a-ENSG00000056736	DGWAS-5707	IL17RB	Coronary heart disease	Inverse variance weighted	3	−0.05	0.03	0.069	0.95
eqtl-a-ENSG00000056736	DGWAS-6504	IL17RB	Coronary atherosclerosis	Inverse variance weighted	3	−0.05	0.03	0.13	0.95
eqtl-a-ENSG00000056736	DGWAS-3027	IL17RB	Coronary atherosclerosis	Inverse variance weighted	3	0.05	0.04	0.23	1.05
eqtl-a-ENSG00000056736	DGWAS-5533	IL17RB	Coronary artery disease	Inverse variance weighted	3	−0.03	0.03	0.26	0.97
eqtl-a-ENSG00000056736	DGWAS-12	IL17RB	Coronary atherosclerosis	Inverse variance weighted	3	0.04	0.04	0.32	1.04
eqtl-a-ENSG00000163702	DGWAS-3027	IL17RC	Coronary atherosclerosis	Inverse variance weighted	2	−0.05	0.03	0.043	0.95
eqtl-a-ENSG00000163702	DGWAS-12	IL17RC	Coronary atherosclerosis	Inverse variance weighted	2	−0.04	0.03	0.18	0.97
eqtl-a-ENSG00000163702	DGWAS-5511	IL17RC	Coronary artery disease	Inverse variance weighted	2	−0.02	0.02	0.27	0.98
eqtl-a-ENSG00000163702	DGWAS-5485	IL17RC	Coronary artery disease	Inverse variance weighted	2	−0.03	0.08	0.72	0.97
eqtl-a-ENSG00000163702	DGWAS-5533	IL17RC	Coronary artery disease	Inverse variance weighted	2	−0.04	0.01	2.50E-08	0.96
eqtl-a-ENSG00000163702	DGWAS-6504	IL17RC	Coronary atherosclerosis	Inverse variance weighted	2	−0.12	0.02	6.00E-09	0.88

The IL-17A, produced by Th17 cells, plays a significant role in the development of cardiovascular diseases like atherosclerosis and is associated with age-related vascular endothelial dysfunction. For example, the proportion of Th17 cells and the expression levels of IL-17A, IL-6, and VCAM-1 increase with age in mice. *In vitro* studies show that IL-17A treatment inhibits the proliferation of mouse aortic endothelial cells (MAECs) and increases the levels of senescent β-galactosidase and senescence-associated proteins (p16, p19, p21, and p53). Blocking the NF-κB pathway with PDTC can inhibit IL-17A-induced expression of senescence-associated proteins. The study reveals a previously unknown link between IL-17A and endothelial cell senescence, mediated by the NF-κB/p53/Rb signaling pathway, suggesting that IL-17A may contribute to endothelial cell senescence and vascular dysfunction with advancing age (Zhang et al., 2021).

The role of genetic knockout mouse models of IL-17A and IL-17RA in cardiovascular diseases has been investigated in literature (Butcher et al., 2012). For instance, studies from (Liao et al., 2012) have shown that IL-17A knockout (IL17A−/−) exhibited a significant reduction in infarct volume after cerebral ischemia/reperfusion (I/R) injury. Similarly, both IL17A−/− and IL-17 receptor A knockout (IL17RA−/−) mice demonstrated protective effects on kidney function and morphology after renal I/R injury. These findings suggest that genetic knockout of IL-17A or its receptor can have beneficial effects in reducing tissue damage in various cardiovascular-related I/R injury models (Liao et al., 2012).

Regarding the use of these mouse models in studies on CMs, there was not explicitly mentioned that have utilized IL-17A or IL-17RA genetic knockout mouse models specifically for investigating the effects of CMs on cardiovascular diseases. However, the potential for such studies exists, as CMs are known for their anti-inflammatory and protective effects on the cardiovascular system, which could be further explored using these genetically modified mouse models to understand their mechanisms of action in modulating IL-17A/IL-17RA pathways in cardiovascular diseases.

IL-17 stimulates macrophages to release pro-inflammatory cytokines, such as TNF-α and IL-6, which are involved in the formation and progression of atherosclerosis (Smith et al., 2010). IL-17 promotes the uptake of lipids and the formation of foam cells by regulating the transition between M1 and M2 macrophage phenotypes. Current treatments related to IL-17 primarily include anti-IL-17 monoclonal antibodies, such as Secukinumab and Ixekizumab. These drugs have been used for autoimmune diseases like psoriasis (Thomas et al., 2024), but their application in patients with CHD requires further exploration. Existing treatments may have side effects and are often non-specific, potentially affecting physiological immune responses. CM has shown promise in regulating inflammation, improving lipid levels, and promoting vascular health, potentially providing new avenues for the treatment of IL-17 and macrophage-related CHD (Ye et al., 2024). CMs such as *Danshen* and *Huangqi* have been found to have positive effects in regulating macrophage function and reducing inflammatory responses.

CM plays an important role in modulating IL-17-regulated macrophages in CHD. CM drug administration may provide potential therapeutic approaches to inhibit vulnerable AS plaques. Although there are few studies reported the role of CM in modulating IL-17-regulated macrophages in CHD, some reports explored and provided useful hints through network pharmacological methods. In terms of mechanism of IL-17-induced inflammation in pro-inflammatory M1 macrophages, CM regulate the polarization of macrophages from M1 type to M2 type, reduce IL-17 production to exert anti-inflammatory effects on the one hand, and reduce LDL oxidation and lipid deposition on the other.

Firstly, regarding the mechanisms of action, Western medicine typically targets specific molecular pathways involved in pathological processes. For example, statins reduce cholesterol synthesis by inhibiting HMG-CoA reductase, thereby decreasing the progression of AS (Kones and Rumana, 2015). Drugs such as aspirin and anti-inflammatory medications primarily focus on inhibiting specific inflammatory pathways, such as the COX-1/COX-2 pathways, directly reducing thrombus formation and inflammatory responses (de Gaetano et al., 2003). Studies indicate that CM plays a crucial role in regulating macrophages, mainly treating AS and preventing CHD through pathways like NF-κB, AMPK, PI3K/AKT, and CCL2/CCR2. TCM formulations such as YDXNT, NXDTD, and monomers like Curcumin, Zedoarondiol, and turmeric nanoparticles (Lipocurc™) exert anti-AS effects by suppressing inflammation in macrophages. CM promotes the polarization of macrophages from M1 to M2, thereby reducing inflammation and stabilizing plaques via CM formulas such as QSG and GGQLD.

Secondly, in terms of cholesterol metabolism regulation, Western medicine mainly modulates lipid metabolism by lowering LDL levels and increasing HDL levels, primarily through statins (Arsenault et al., 2011). In contrast, CM reduces the progression of CHD by promoting cholesterol efflux and inhibiting foam cell formation in macrophages. For instance, WDD, YQHXF, and their monomers Formononetin and Salviain achieve this by decreasing the expression of LDL-related receptors or upregulating the expression of cholesterol efflux-related receptors on macrophages, thus altering plaque composition, reducing monocyte adhesion, and inflammation, fundamentally delaying the progression of AS.

Finally, regarding the regulation of inflammatory pathways, Western medicine usually intervenes by targeting specific inflammatory mediators. For example, corticosteroids broadly suppress immune responses, which may lead to immune suppression and side effects (Vinet et al., 2015). In contrast, CMs such as BYHWD and YQHXF regulate the IL-17 pathway, reducing the expression of inflammatory factors in AS and thereby decreasing the risk of its formation. Moreover, some CMs stabilize plaques by modulating macrophage ferroptosis.

In summary, CM shows unique advantages in regulating macrophage function, promoting polarization, and modulating cholesterol metabolism, providing a more complex and comprehensive therapeutic mechanism compared to Western medicine. This capability can improve and stabilize the atherosclerotic process. These characteristics give CM significant development potential in treating coronary heart disease and atherosclerosis and may serve as an effective complement to Western pharmacotherapy. However, the current understanding of the mechanisms of CM is still incomplete, necessitating more clinical and basic research to validate its safety and efficacy.

## 6 Limitation and prospective

The current review underscores the potential of CM in modulating IL-17-regulated macrophages for treating CHD, but it also exists several limitations. Key issues include the limited clinical data, as most studies focus on *in vitro* experiments and animal models, necessitating further validation through well-designed clinical trials. Additionally, the heterogeneity in study designs and the lack of standardized methods for defining macrophage subtypes complicate result comparison and interpretation. While significant progress has been made in understanding how specific CM formulas regulate macrophage subpopulations, most research has concentrated on short-term effects, with insufficient long-term data on effectiveness and safety. The limitations of this study include the fact that it primarily focuses on M1/M2 macrophages. Recent scRNA-sequencing studies of human and mouse atherosclerotic plaques have unveiled the existence of macrophages with characteristics that do not only fit into the traditional M1 or M2 categories (Li Q. et al., 2022). By concentrating solely on M1/M2 macrophages, it may potentially limit the comprehensive understanding of the inflammatory processes and the full spectrum of macrophage phenotypes involved in the development and progression of cardiovascular diseases (Chaudhry et al., 2019; Yu et al., 2023). Thus, further studies should also pay attention to the contributions and roles of these non-M1/M2 macrophages in the pathogenesis of cardiovascular diseases such as AS. Addressing these gaps will require comprehensive research efforts, including standardized methodologies and broader evaluations of CM interventions for CHD. Similarly, Western medicine research on IL-17 and macrophage mechanisms also faces challenges, such as insufficient target specificity of therapies and a primary focus on short-term efficacy without long-term assessments. Moreover, individual variability in responses to IL-17 inhibitors and the complexity of immune interactions are often overlooked. The reliance on animal models further limits the clinical applicability of findings. These challenges highlight the need for more foundational and clinical research in both CM and Western medicine to enhance our understanding and improve therapeutic outcomes for CHD.

Based on the gaps identified in the review, several specific research areas can be explored to deepen our understanding of CM in modulating IL-17-regulated macrophages for the treatment of CHD.

First, it is essential to conduct large-scale, double-blind, placebo-controlled randomized controlled trials (RCTs) to evaluate the efficacy and safety of specific CM formulas and combinations in treating CHD. Comparative studies should assess the effectiveness and safety of CM interventions against conventional treatments, such as statins or anti-inflammatory medications. Longitudinal studies are needed to investigate the long-term effects of CM interventions on plaque stability, cardiovascular events, and quality of life. Additionally, exploring how individual factors—such as genetics, gut microbiota, and lifestyle—affect responses to CM interventions could pave the way for personalized medicine.

Second, mechanism studies should focus on the cellular and molecular pathways through which CM regulates IL-17 and modulates macrophages. Utilizing network pharmacology can help identify potential synergistic effects between various CM compounds and their molecular targets. Establishing animal models will enable investigation into the effects of CM on plaque formation and stability in CHD models. It's also crucial to assess how CM interventions influence the immune system, including the balance between Th17 and Treg cells and the activation of regulatory T cells.

Finally, exploring other avenues, such as the synergistic effects of combining CM with lifestyle therapies (like exercise, nutrition, or mindfulness), could yield valuable insights for enhancing treatment outcomes. Comprehensive safety and toxicity studies are necessary to evaluate the potential adverse effects of CM interventions. Additionally, research into the absorption, distribution, metabolism, and excretion of CM compounds can help optimize dosing strategies.

## 7 Conclusion

The findings of this review suggest that CM holds promise as a valuable therapeutic approach to modulate IL-17-regulated macrophages in CHD. However, its mechanisms of action and clinical efficacy require further investigation. With appropriate research and implementation, TCM could play an important role in improving CHD management and enhancing patient outcomes.
